# Farnesoid X receptor, overexpressed in pancreatic cancer with lymph node metastasis promotes cell migration and invasion

**DOI:** 10.1038/bjc.2011.37

**Published:** 2011-03-01

**Authors:** J Y Lee, K T Lee, J K Lee, K H Lee, K-T Jang, J S Heo, S H Choi, YIl Kim, J C Rhee

**Affiliations:** 1 Samsung Biomedical Research Institute, Seoul, Korea; 2 Department of Medicine, Samsung Medical Center, 50 Irwon-dong, Gangnam-gu, Sungkyunkwan University School of Medicine, Seoul 135-710, Korea; 3 Department of Pathology, Sungkyunkwan University School of Medicine, Seoul, Korea; 4 Department of Surgery, Samsung Medical Center, Sungkyunkwan University School of Medicine, Seoul, Korea

**Keywords:** pancreatic cancer, lymph node metastasis, FXR, DNA microarray, siRNA

## Abstract

**Background:**

Lymph node metastasis is one of the most important adverse prognostic factors for pancreatic cancer. The aim of this study was to identify novel lymphatic metastasis-associated markers and therapeutic targets for pancreatic cancer.

**Methods:**

DNA microarray study was carried out to identify genes differentially expressed between 17 pancreatic cancer tissues with lymph node metastasis and 17 pancreatic cancer tissues without lymph node metastasis. The microarray results were validated by real-time PCR. Immunohistochemistry and western blotting were used to examine the expression of farnesoid X receptor (FXR). The function of FXR was studied by small interfering RNA and treatment with FXR antagonist guggulsterone and FXR agonist GW4064.

**Results:**

Farnesoid X receptor overexpression in pancreatic cancer tissues with lymph node metastasis is associated with poor patient survival. Small interfering RNA-mediated downregulation of FXR and guggulsterone-mediated FXR inhibition resulted in a marked reduction in cell migration and invasion. In addition, downregulation of FXR reduced NF-*κ*B activation and conditioned medium from FXR siRNA-transfected cells showed reduced VEGF levels. Moreover, GW4064-mediated FXR activation increased cell migration and invasion.

**Conclusions:**

These findings indicated that FXR overexpression plays an important role in lymphatic metastasis of pancreatic cancer and that downregulation of FXR is an effective approach for inhibition of pancreatic tumour progression.

Pancreatic cancer is one of the most clinically aggressive malignancies, with a 3-year survival rate of only 17% after surgical resection of the primary tumour (Niederhuber *et al*, 1995). Poor prognosis of pancreatic cancer is related with early metastasis to regional lymph nodes (LNs) or liver, as the presence or absence of LN metastasis is an important prognostic factor for patients with pancreatic cancer (Mao *et al*, 1995; Maeda *et al*, 2007). However, the mechanisms involved in LN metastasis of pancreatic cancer are not fully understood. Therefore, identification of molecular marker that might predict LN metastasis of pancreatic cancer is important in selecting patients who would benefit from surgical treatment or molecular targeting therapy.

The farnesoid X receptor (FXR) is a member of the nuclear receptor superfamily of ligand-dependent transcription factors that forms a heterodimer with the retinoid X receptor (Forman *et al*, 1995; Seol *et al*, 1995). Farnesoid X receptor is highly expressed in the liver, gut, kidney, and adrenal cortex, and regulates a variety of genes with roles in bile acid homeostasis, lipid, and glucose metabolism (Laffitte *et al*, 2000; Wang *et al*, 2008). In contrast, low levels of mRNA for FXR are present in a variety of tissues, including heart, ovary, thymus, eye, spleen, and testes (Otte *et al*, 2003; Bishop-Bailey *et al*, 2004). The roles of FXR in these tissues are poorly understood, particularly within human beings. Recently, FXR was reported to play a role in tumour growth. Farnesoid X receptor-null mice spontaneously develop a high incidence of liver tumours, including hepatocellular adenoma, carcinoma, and hepatocholangiocellular carcinoma (Kim *et al*, 2007). In addition, FXR has recently been detected in breast cancer tissue and breast cancer cell lines (Silva *et al*, 2006; Swales *et al*, 2006; Journe *et al*, 2008). The correlation between FXR expression and tumour cell proliferation suggests that, in addition to the involvement of FXR in cell survival and proliferation, this nuclear receptor is involved in tumour invasiveness and metastasis (Journe *et al*, 2009). Farnesoid X receptor is expressed in colorectal tumour cells; however, it is not clear how bile acid-induced activation of FXR triggers carcinogenesis or tumour metastasis (De Gottardi *et al*, 2004).

In this study, we have characterised for the first time the role of FXR in pancreatic cancer. These studies began with microarray analyses of pancreatic cancers with LN metastasis *vs* those without LN metastasis in tumours of the same size. One of the most interesting candidate genes identified by this approach, FXR, was selected for further investigation for its expression. We examined the consequence of FXR siRNA, guggulsterone (GS), and GW4064 treatment on pancreatic cancer proliferation, migration, and invasion.

## Materials and methods

### Clinical specimens

A total of 34 clinical pancreatic cancer tissues were obtained from surgical resection collected at Samsung Medical Center. We divided the samples into two groups according to the absence (Group I) or presence (Group II) of LN metastasis, and the clinicopathological characteristics of tumour stage and survival dates of each group are summarised in Table 1.

The cancer stage of each case was classified according to the tumour node metastasis (TNM) classification of the Union International Contre le Cancer. Tumour node metastasis was defined as size or direct extent of the primary tumour (T, 1–4) that spreads to regional LNs (N, 0–3) and develops distant metastasis (M, 0/1). Clinical follow-up was obtained from patient's medical record. Postoperative survival was measured from the day of surgery to the death or censored last follow-up visit. The mean patient survival in Group I without LN metastasis was 23.4±8.8 months, whereas the mean patient survival in Group II with LN metastasis was 9.1±3.9 months. Most of the clinicopathological characteristics of the two groups, except for LN metastasis, were similar (i.e., surgical method, degree of differentiation of cancer, tumour size, and the absence of distant metastasis).

Informed consent was obtained from all patients, and this study was approved by the Samsung Medical Center Institutional Review Board. Tissue samples were frozen in liquid nitrogen immediately after surgical removal and were maintained at a temperature of −80°C until required for RNA extraction. From each frozen tissue sample, serial sections were made and stained with haematoxylin and eosin. A pathologist reviewed the serial slides and selected representative sections that contained >70% of ductal adenocarcinomas in the tissue section volume.

### Isolation of total RNA, gene expression array, and analysis of microarray data

Human genome survey arrays (Applied Biosystems, Foster City, CA, USA) were used to analyse the transcriptional profiles of the pancreatic cancer tissue RNA samples. RNA isolation, sample preparation, and array hybridisation were performed as described previously (Kim *et al*, 2010a). The Applied Biosystems Expression System software (Applied Biosystems) was used to extract assay signals and assay signal-to-noise ratios from the microarray images. The bad spots flagged by the software were removed from the analysis. The raw data were exported to Excel files (Microsoft Corporation, Redmond, WA, USA). The exported data files were analysed using Avadis software (StrandGenomics, Bangalore, India) and Arrayassist (Stratagene, La Jolla, CA, USA). The assay signals of the remaining set of 99.5% of the genes were log-transformed. To select differentially expressed genes, the remaining sets of 99.5% of the genes were further filtered by means of a standard expression array system signal-to-noise threshold (the signal-to-noise ratio was >3 in at least one sample) and a flag threshold (<5000). The filtered genes were normalised using the quantile normalisation method. Significant genes were calculated using one-way analysis of variance and the Tukey’s HSD test (*P*<0.05) and the fold-change method. Statistical significances were adjusted by Benjamini–Hochberg FDR multiple-testing correction. Fold change was calculated using the average of each group.

### Real-time PCR for gene expression studies

Real-time analysis for target genes was performed as described previously (Kim *et al*, 2010a).The TaqMan probe/primer sets were as follows: LZIC, Hs00260696_m1; FXR, Hs00231968_m1; SCAMP1, Hs00191607_m1; and SULT1E1, Hs00193690_m1. The target amount was then divided by an endogenous reference amount (GAPDH; Applied Biosystems) to obtain a normalised target value.

### Reverse transcription–PCR analysis

Total RNA was prepared from cells using the RNeasy Mini kit (Qiagen, Chatsworth, CA, USA) according to the manufacturer's instructions. Total RNA was converted into single-strand cDNA with the Moloney murine leukaemia virus RT (Invitrogen, Grand Island, NY, USA) with random hexamer primers. Primers for human small heterodimer partner (SHP) and glyceraldehyde-3-phosphate dehydrogenase (GAPDH) were as described in Kim *et al* (2010b).

### Tissue material and immunohistochemistry

Tissue sections on glass slides were deparaffinised with xylene, rehydrated in serially diluted alcohol, and subsequently processed in a microwave for 15 min with Tris-EDTA (TE; pH 9) buffer for antigen retrieval. After blocking of endogenous peroxidase with 3% H_2_O_2_, the sections were immersed in 3% goat serum diluted with phosphate-buffered saline for 60 min. The slides were then incubated with a mouse monoclonal anti-human FXR antibody (1:30 dilution; R&D Systems Co., Minneapolis, MN, USA) for 90 min at room temperature. After rinsing three times with distilled water containing 0.1% Tween-20, the tissue sections were incubated with HRP-conjugated streptavidin for 20 min at room temperature. Slides were then washed, developed for 5 min with liquid 3,3′-diaminobenzidine tetrahydrochloride, counterstained with Meyer's haematoxylin, dehydrated, and mounted with Permount for histological examination. The results of immunostaining were recorded as an intensity score according to the estimated staining proportion (no, 0; weak, <10% moderate, 10∼50% and strong, >50%).

### Cell lines and reagents

Human pancreatic cancer cell lines (MIA-PaCa2, PANC-1, AsPC-1, Capan-1 and Capan-2), the HepG2 hepatoma cell line, and the MCF-7 breast cancer cell line used in this study were obtained from the American Type Culture Collection (Manassas, VA, USA). GS, GW4064 and mitomycin C were purchased from Sigma (St Louis, MO, USA).

### Downregulation of FXR expression by siRNA

Cells were transfected with FXR sequence and control siRNA using Lipofectamine RNAiMAX reagent (Invitrogen) according to the manufacturer's instructions. After transfection at a final concentration of 30 nM siRNA, cells were cultured for 72 h. The sequence targeting human FXR for siRNA (5′-GAGGAUGCCUCAGGAAAUA-3′) was synthesised and annealed by Invitrogen. The control siRNA (scramble; 5′-AAAGCGUCUGGAAAAGUCG-3′) from Invitrogen was used to evaluate the nonspecific effects on transfection on gene expression.

### Western blot analysis

Fifty micrograms of proteins were separated on NuPAGE Novex Bis–Tris 4–12% gels (Invitrogen) and electroblotted onto nitrocellulose membranes. Membranes were then incubated in blocking solution (5% milk in 20 mM Tris HCl, 150 mM NaCl, and 0.1% Tween-20), followed by overnight incubation with a mouse monoclonal anti-human FXR antibody (diluted 1:250; R&D Systems). Peroxidase-labelled anti-mouse IgG antibody (1:10 000; Cell Signaling, Beverly, MA, USA) was used as a secondary reagent. Bound peroxidase activity was revealed using the SuperSignal West Pico Chemiluminescent Substrate (Pierce, Rockford, IL, USA).

### Determination of cell proliferation

Cell proliferation was determined by the Dojindo Cell Counting Kit-8 (Dojindo, Gaithersburg, MD, USA). This assay is based on the cleavage of the tetrazolium salt WST-8 by mitochondrial dehydrogenase in viable cells (Aghdassi *et al*, 2007). Cells were seeded in 96-well plates at a density of 5 × 10^3^ cells in 100 *μ*l of culture medium and allowed to adhere overnight. After FXR siRNA transfection or treatment with GS, 10 *μ*l of the tetrazolium substrate were incubated at 37°C for 1 h and the absorbance at 450 nm was measured.

### Cell migration and invasion assay

Cell migration was assessed using 24-well inserts (Becton Dickinson Labware, Franklin Lakes, NJ, USA) with 8-*μ*m pores according to the manufacturer's protocol. After 24 h of incubation, the cells in the upper chamber were removed, and the cells were fixed in ice-cold methanol, stained with Wright–Giemsa solution (Polysciences, Warrington, PA, USA). Digital images were obtained from the membranes, and cell areas were selected using Scan Scope CS system (Aperio Technologies, Vista, CA, USA). The migrating cells were quantified in five randomly selected fields at × 40 magnification in each membrane, and the average value was defined as a migration or invasion index on three independent membranes. Studies of invasion were performed as described earlier, except that the membranes utilised were Matrigel-coated invasion chambers (BD Biosciences, Bedford, MA, USA) that were pre-hydrated in serum-free medium.

### Enzyme-linked immunosorbent assay

Activation of NF-*κ*B p65 was evaluated by enzyme-linked immunosorbent assay (ELISA) (Active Motif, Carlsbad, CA, USA) on nuclear extracts prepared with the nuclear extract kit (Active Motif) according to the manufacturer's instruction (de Haij *et al*, 2003; Van Acker *et al*, 2007). The culture medium of the FXR siRNA-transfected MIA-PaCa2 and PANC-1 cells assayed immediately using commercially available VEGF ELISA kits (R&D Systems).

### Densitometric and statistical analysis

The bi-dimensional optical densities of FXR and *β*-actin proteins on the films were quantified and analysed with Molecular Analyst software (Bio-Rad, Hercules, CA, USA). Results are expressed as mean±s.e.m. Group differences were statistically analysed using the Student's *t-*test. Survival curves were calculated by the Kaplan–Meier method and differences were analysed by the log-rank test. Correlations between FXR expression and LN metastasis were analysed using the Mann–Whitney *U*-test. The Kaplan–Meier method was used to generate survival curves, and differences in probability values <0.05 were considered statistically significant.

## Results

### Identification of genes differentially expressed between pancreatic cancer tissues with and without LN metastasis

To minimise the number of falsely significant genes, genes in which the signal-to-noise ratio was >3 in at least one sample and the flag value was <500 074 were filtered, leaving 15 357 from the original 30 469 genes. Of the 15 357 genes that were analysed on the cDNA arrays, 184 genes (*t-*test, *P*<0.05) were differentially expressed between pancreatic cancer tissues with LN metastasis and those without LN metastasis. For the two groups we analysed, only 58 genes were differentially expressed, with the difference in signal intensity ratio >1.5 fold change. Of these genes sets, 15 genes were significantly upregulated in pancreatic cancer tissues with LN metastasis compared with those without LN metastasis. Table 2 contains complete lists of upregulated genes in pancreatic cancer tissues with LN metastasis with the name, description and chromosome location.

### Validation of microarray data by real-time PCR

**Figure 1:**
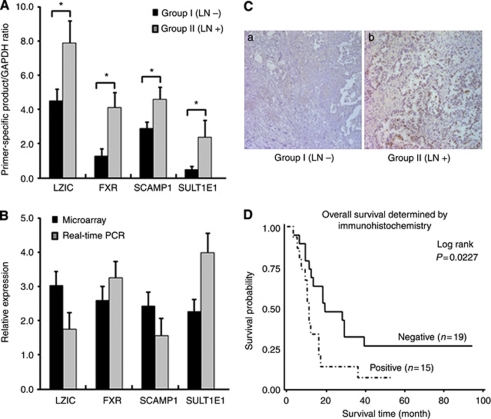
Gene expression analysis of selected upregulated genes and immunohistochemical expression of FXR in pancreatic cancer tissues with and without LN metastasis. (**A**) Upregulated gene analysis between pancreatic cancer tissues with and without LN metastasis by real-time PCR. (**B**) Real-time PCR validation of microarray data. For each sample, the amount of target and endogenous reference was determined from the appropriate standard curve. The target amount was then divided by an endogenous reference (GAPDH) amount to obtain a normalised target value. The black columns represent the mean of fold (Group II/Group I) values obtained in DNA microarray; the grey columns represent the mean of fold (Group II/Group I) values obtained in real-time PCR; ^*^P<0.05. (**C**) Overexpression of FXR in pancreatic cancer tissues with LN metastasis. The FXR intensity on tissues was evaluated by immunohistochemical staining. (a) A representative negative staining in pancreatic cancer tissues without LN metastasis. Magnification, × 200. (b) A representative positive staining in pancreatic cancer tissues with LN metastasis. Magnification, × 200. (**D**) Kaplan–Meier analysis of overall survival for patients with ductal adenocarcinoma (*n*=34). Farnesoid X receptor expression-positive cases (*n*=15) had significantly poorer prognosis than expression -negative cases (*n*=19; *P*=0.0227, log-rank test).

The differences in gene expression found by microarray analyses were validated by real-time PCR. We chose genes that were >2-fold upregulated according to our microarray results (Table 2). Among the genes, we evaluated the levels of expression of four selected upregulated genes in pancreatic cancer tissues with LN metastasis. The expression of LZIC, FXR, SCAMP1, and SULT1E1 was significantly higher in pancreatic cancer tissues with LN metastasis than in pancreatic cancer tissues without LN metastasis (Figure 1A). Although some variation concerning the degree of regulation was observed, the data obtained with microarrays were substantially confirmed for four selective genes by real-time PCR (Figure 1B). In summary, microarrays together with real-time PCR validation results clearly show that the expression of LZIC, FXR, SCAMP1, and SULT1E1 were confirmed to be significantly higher in pancreatic cancer tissues with LN metastasis than in pancreatic cancer tissues without LN metastasis. These results are representative for measurements accomplished with pancreatic cancer tissues from three different experiments.

### Immunohistological analysis of FXR expression

Among upregulated genes, FXR was selected for further investigation because FXR, as a therapeutic target, has never been studied in pancreatic cancer. To confirm the overexpression of FXR protein in pancreatic cancer tissues with LN metastasis, we performed immunohistochemical staining using anti-FXR antibody and validated overexpression of FXR protein in pancreatic cancer tissues with LN metastasis (Figure 1C). For further analysis, the immunostaining results were grouped into two categories. Those that exhibited >10% immunoreactivity were classified as positive, whereas those with no or <10% immunoreactivity was classified as negative. We observed that 70.6% (12 of 17) of pancreatic cancer tissues with LN metastasis were positive, of which staining was strong in 17.6%, moderate in 29.4%, and weak in 23.5% (Figure 1Cb). In pancreatic cancer tissues without LN metastasis, positive expression of FXR was found in 17.6% (3 of 17) and negative in 82.4% (14 of 17) (Figure 1Ca). Overall, staining was determined to be significantly stronger in pancreatic cancer tissues with LN metastasis than in pancreatic cancer tissues without LN metastasis for FXR expression (*P*=0.0044). Overall survival analysis using the Kaplan–Meier method revealed that the prognosis of patients with tumours expressing positive FXR was significantly poorer than that with tumours expressing negative FXR (Figure 1D; *P*=0.0227 by log-rank test). These findings are consistent with the hypothesis that FXR-expressing cancers are somewhat more aggressive than FXR-negative cancers, but only if other adverse prognostic factors do not result in a rapid postoperative death. Subsequently, we scanned several human pancreatic adenocarcinoma cells for the expression of FXR.

### Downregulation of FXR expression by siRNA inhibited cell proliferation and decreased cell migration and invasion

**Figure 2:**
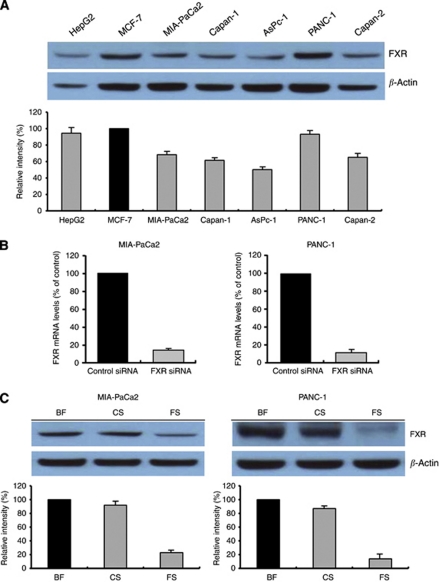
Farnesoid X receptor expression in pancreatic cancer cell lines. BF, basal FXR; CS, control siRNA; FS, FXR siRNA. (**A**) Western blot analysis of FXR was carried out on 50 *μ*g of total proteins extracted from five pancreatic cancer cell lines; HepG2 and MCF-7 cells seen as a band at ∼56 kDa; HepG2 and MCF-7 were used as positive controls. *β*-Actin was used as a loading control. (**B**, **C**) MIA-PaCa2 and PANC-1 cells were transfected with control siRNA or FXR siRNA for 72 h. (**B**) Total RNA was extracted and analysed by TaqMan real-time quantitative RT–PCR. Farnesoid X receptor mRNA values were normalised to GAPDH mRNA. (**C**) Total proteins were extracted and western blotting analysis was performed. *β*-Actin was used as a loading control. Band intensities were evaluated in terms of relative density and expressed as percentages of the control, which was assumed to be 100%. Columns, mean of three independent experiments; bars, s.e.m.

The baseline expression of FXR was determined in a panel of human pancreatic cancer cell lines that included AsPC-1, Capan-1, Capan-2, MIA-PaCa2, and PANC-1. The results showed that FXR was frequently but differentially expressed in different human pancreatic cancer cell lines (Figure 2A). HepG2 and MCF-7 cells were used as positive controls for FXR expression (Bishop-Bailey *et al*, 2004; Swales *et al*, 2006).

To determine whether or not FXR could be an effective therapeutic target for pancreatic cancer, the effect of FXR siRNA on cell proliferation was examined in MIA-PaCa2 and PANC-1 cells. The efficacy of FXR siRNA for knock down of FXR mRNA and protein was confirmed by real-time RT–PCR and western blotting, respectively. We observed that both FXR mRNA and protein levels were barely detectable in FXR siRNA-transfected cells compared with siRNA control-transfected cells (Figures 2B and C).

**Figure 3:**
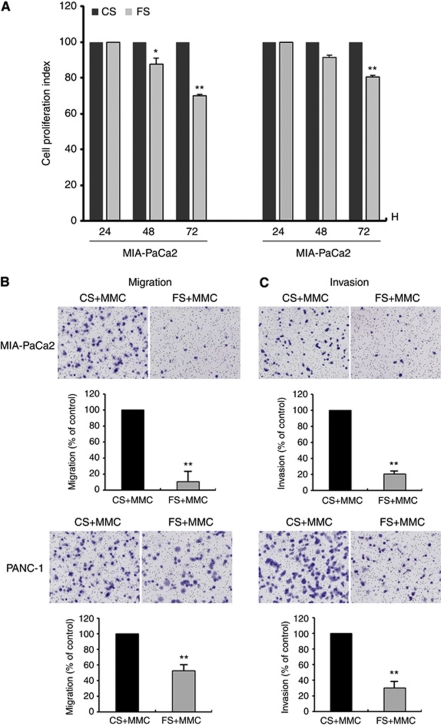
Effects of FXR siRNA on pancreatic cancer cell proliferation, migration, and invasion. BF, basal FXR; CS, control siRNA; FS, FXR siRNA; MMC, mitomycin C. (**A**) MIA-PaCa2 and PANC-1 cells were transfected with control siRNA or FXR siRNA for the indicated time periods before cell number were estimated using CCK-8 assay. (**B**, **C**) MIA-PaCa2 and PANC-1 cells transfected with control siRNA or FXR siRNA were cultured in the presence of MMC (μg ml^−1^) for 48 h. Cell were placed in serum-free culture media and added into the upper compartment of a migration or invasion chamber. After 24 h, cells in the upper chamber were removed and cells that had migrated (**B**) or invaded (**C**) onto the lower surface of the membrane were fixed and stained with Wright–Giemsa. The relative-fold migration and invasion values of FXR siRNA-transfected cells was normalised against control siRNA-transfected cells and expressed as percentages of the control, which was assumed to be 100%. Columns, mean of three independent experiments; bars, s.e.m. ^**^P<0.01.

The cell proliferation was determined by CCK-8, and the effect of FXR siRNA on the proliferation of cancer cells is shown in Figure 3A. We found that the downregulation of FXR expression inhibited cell proliferation in both pancreatic cancer cell lines. The cell migration and invasion assay were performed with a proliferation blocker (mitomycin C) to observe the effect of FXR knockdown on the cell migratory or invasive potential without an effect on cell proliferation. We found that downregulation of FXR decreased cell migration (Figure 3B). Moreover, FXR siRNA-transfected cells showed a low level of penetration through the Matrigel-coated membrane compared with the control cells (Figure 3C).

### Downregulation of FXR decreased NF-*κ*B DNA-binding activity and VEGF activity

**Figure 4:**
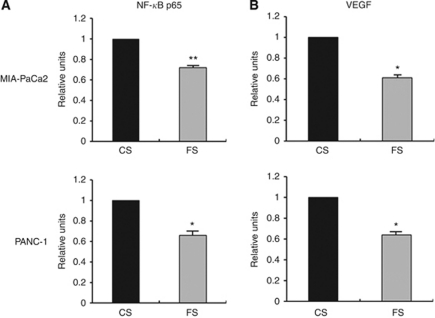
Effects of FXR siRNA on NF-*κ*B p65 and VEGF activities. CS, control siRNA; FS, FXR siRNA. (**A**) Nuclear extracts were prepared from control siRNA or FXR siRNA-transfected MIA PaCa2 and PANC-1 cells and subjected to analysis for NF-*κ*B p65 activity as measured by Active Motif enzyme-linked immunosorbent assay (ELISA). (**B**) The culture medium of control siRNA or FXR siRNA-transfected MIA-PaCa2 and PANC-1 cells was used for the detection of VEGF using ELISA, as described under Materials and Methods. The relative-fold change of NF-*κ*B p65 or VEGF activity in FXR siRNA-transfected cells was normalised against control FXR siRNA-transfected cells. Values in control siRNA-transfected cells were arbitrarily set to 1. Columns, mean of three independent experiments; bars, s.e.m. ^*^P<0.05; ^**^P<0.01.

The NF-*κ*B signalling pathway is involved in cancer cell migration, invasion, and metastasis processes. Therefore, we measured the NF-*κ*B DNA-binding activity in transfected pancreatic cancer cells. Nuclear extracts from control and FXR siRNA-transfected pancreatic cancer cells were subjected to analysis for NF-*κ*B DNA-binding activity, as measured by ELISA. It was found that downregulation of FXR by siRNA transfection decreased NF-*κ*B DNA-binding activity (Figure 4A). The expression of VEGF is regulated by NF-*κ*B and has been reported to play an important role in tumour progression (Zeng *et al*, 2003; Xiong *et al*, 2004). To further explore whether FXR siRNA reduced VEGF activity, we examined the levels of VEGF activity secreted in the culture medium. We found that downregulation of FXR could lead to a decrease in the levels of VEGF secreted in the culture medium (Figure 4B).

### GS inhibited cell proliferation and decreased migration and invasion

**Figure 5:**
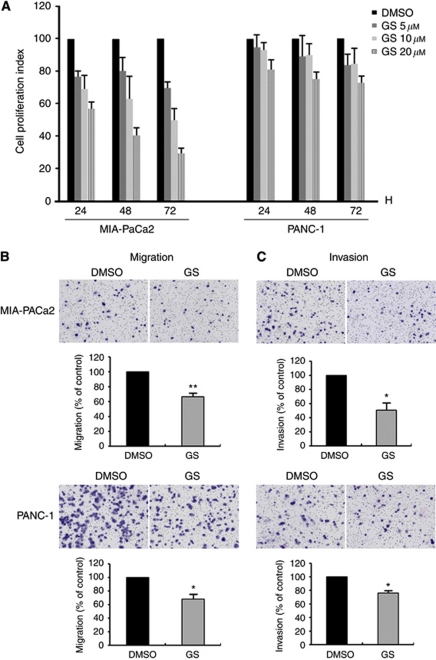
Effects of FXR antagonist guggulsterone on pancreatic cancer cell proliferation, migration, and invasion. DMSO, control; GS, guggulsterone. (**A**) MIA-PaCa2 and PANC-1 cells were treated with 5, 10, and 20 μM GS or DMSO for the indicated time periods before cell numbers were estimated using CCK-8 assay. (**B**, **C**) MIA-PaCa2 and PANC-1 cells were treated with 10 μM GS for 48 h. Cells were placed in serum-free culture media and added into the upper compartment of a migration or invasion chamber. After 24 h, cells in the upper chamber were removed and cells that had migrated (**B**) or invaded (**C**) onto the lower surface of the membrane were fixed and stained with Wright–Giemsa. The relative-fold migration and invasion values of GS-treated cells were normalised against DMSO-treated cells and expressed as percentages of the control, which was assumed to be 100%. Columns, mean of three independent experiments; bars, s.e.m. ^*^P<0.05; ^**^P<0.01.

We examined whether chemical inhibition of FXR affects cancer cell proliferation, migration, and invasion. In both cell lines, treatment with GS inhibited cell proliferation in a dose-dependent manner (Figure 5A). GS (10 μM) inhibited cell proliferation in MIA-PaCa2 and PANC-1 cells compared to cells not treated with GS that were observed after 72 h. We also found that both MIA-PaCa2 and PANC-1 cells decreased cell migration and invasion by treatment with 10 μM GS (Figures 5B and C).

### GW4064 increased cell migration and invasion

**Figure 6:**
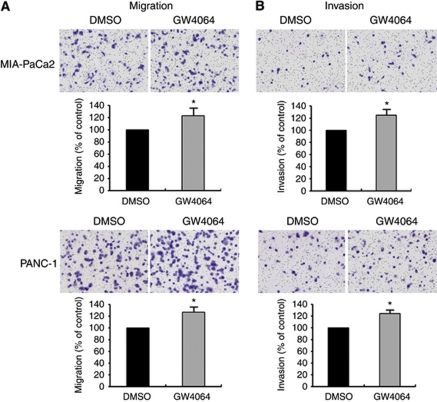
Effect of FXR agonists GW4064 on pancreatic cancer cell migration and invasion. DMSO, control. (**A**, **B**) MIA-PaCa2 and PANC-1 cells were treated with 1 μM GW4064 for 48 h. Cells were placed in serum-free culture media and added into the upper compartment of a migration or invasion chamber. After 24 h, cells in the upper chamber were removed and cells that had migrated (**A**) or invaded (**B**) onto the lower surface of the membrane were fixed and stained with Wright–Giemsa. The relative-fold migration and invasion values of GW4064-treated cells were normalised against DMSO-treated cells and expressed as percentages of the control, which was assumed to be 100%. Columns, mean of three independent experiments; bars, s.e.m. ^*^P<0.05.

To further confirm the role of FXR on cell migration and invasion, cells were treated with GW4064, a selective and strong FXR agonist (Maloney *et al*, 2000; Martinez-Fernandez *et al*, 2009). We observed that both MIA-PaCa2 and PANC-1 cells increased cell migration and invasion by treatment with 1 μM GW4064 (Figures 6A and B). In addition, to explore a correlation with FXR target gene, SHP (Goodwin *et al*, 2000), we performed RT–PCR. We observed that SHP mRNA expression was very low in MIA-PaCa2 and PANC-1 cells compared with high levels detected in the HepG2 cell lines, used as a positive control. However, GW4064 did not induce an increase of SHP mRNA in both cell lines (data not shown).

## Discussion

Farnesoid X receptor is important in the progression of several human cancers (De Gottardi *et al*, 2004; Silva *et al*, 2006; Swales *et al*, 2006; Kim *et al*, 2007; Journe *et al*, 2008, 2009). However, the expression of FXR and its role in human pancreatic cancer has not been investigated previously. Our study showed, for the first time, that FXR is highly expressed in human pancreatic adenocarcinoma specimens and in five different human pancreatic cancer cell lines tested, suggesting that FXR could be important in human pancreatic cancer progression. Indeed, microarray analysis, together with real-time PCR validation results, clearly showed hat FXR was upregulated in pancreatic cancer tissues with LN metastasis. We also found that FXR overexpression in pancreatic cancer tissues with LN metastasis was associated with poor patient survival. Therefore, these results suggest that FXR overexpression could be a useful prognostic marker for patients with pancreatic cancer.

The role of FXR in growth regulation, apoptosis, and cancer is still controversial. Several studies have established both positive and reciprocal correlations between FXR expression and cancer (De Gottardi *et al*, 2006; Kim *et al*, 2007; Journe *et al*, 2008; Modica *et al*, 2008; Maran *et al*, 2009). However, the precise role and mechanism of FXR for tumour cell proliferation, migration, and invasion remains unclear. In this study, we have shown the following: (a) downregulation of FXR by siRNA inhibited cell proliferation and decreased migration and invasion; (b) downregulation of FXR reduced NF-*κ*B DNA-binding activity and VEGF activity; (c) the FXR antagonist GS inhibited cell proliferation and decreased migration and invasion; and (d) the FXR agonist GW4064 increased cell migration and invasion. Taken together, these results suggest that the downregulation of FXR is an effective approach for the inactivation of FXR and downregulation of its target genes, such as NF-*κ*B and VEGF, resulting in the inhibition of invasion and metastasis of pancreatic cancer cells.

Although the underlying mechanism is not clear, FXR may be involved in regulating cell proliferation, migration, and invasion by multiple mechanisms in several cancer types. In breast cancer, significant correlations have been observed between the expression of FXR and proliferation markers (Ki-67 and several other proliferation markers), including TopoII and c-myc transcription factors (Journe *et al*, 2008). It has been reported that GS decreases the expression of gene products involved in proliferation (cyclin D1 and c-myc) by inhibition of NF-*κ*B and I*κ*B*α* kinase activation in human cells derived from lung carcinoma and leukaemia (Shishodia and Aggarwal, 2004). A recent study shows that Z-GS blocks the proliferation of human tumour cell types, including leukaemia, head and neck carcinoma, multiple myeloma, lung carcinoma, melanoma, breast carcinoma, and ovarian carcinoma by arresting the cells in the S-phase of the cell cycle (Shishodia *et al*, 2007). Similar observations have been made in other malignancies. A previous study has shown that bile acid-sensitive PCmsrc and HCT-8/E11 human colorectal cancer cell lines expressed FXR, and bile acids stimulated invasion through activation of multiple oncogenic signalling (Debruyne *et al*, 2002). In addition, the FXR antagonist, Z-GS, prevented the migration of metastatic human breast cancer MDA-MB-231 cells (Silva *et al*, 2006).

Indeed, we found that transfection with FXR siRNA and treatment with GS resulted in a marked reduction in cell proliferation, migration, and invasion, suggesting that FXR expression promote tumour progression in pancreatic cancer. Moreover, this study revealed that FXR activation by GW4064 increased cell migration and invasion. On the basis of our results, we could speculate FXR as a tumour progressor in pancreatic cancer.

However, several other studies showed FXR as a tumour suppressor rather than a tumour progressor. Modica *et al* (2008) have shown that loss of FXR in mouse models of intestinal tumorigenesis results in early mortality and increased tumour progression. Farnesoid X receptor deficiency has been shown to increase adenocarcinoma size in the small intestine of the APC^min^ mice and increase the prevalence and size of AOM-induced adenocarcinoma in the colon (Maran *et al*, 2009).

Nuclear factor-*κ*B activation is closely involved in the progression of pancreatic cancer due to its ability to increase expression of angiogenic factors including VEGF, and promote the migration and invasion of pancreatic cancer cells (Yebra *et al*, 1995; Xiong *et al*, 2004).

Angiogenesis has been known to play an important role in the development of tumour growth and LN metastasis. Vascular endothelial growth factor potently increases vascular permeability and promotes the formation of new blood vessels in tumour, and thus is regarded as the main growth-stimulatory factor in the tumour-related angiogenesis (Lequerica-Fernandez *et al*, 2007). The prognostic value of high expression of VEGF has been shown in various types of human tumours (Arinaga *et al*, 2003; Tamura *et al*, 2004; Lequerica-Fernandez *et al*, 2007). It has been reported that pancreatic cancer patients with LN metastasis have higher serum concentrations of VEGF when compared with those without LN involvement (Karayiannakis *et al*, 2003). High level of VEGF expression significantly correlated with advanced tumour stage and LN metastasis (Sun *et al*, 2007). Investigations by other laboratories have shown that VEGF promotes migration and invasion of pancreatic cancer cells (Fujioka *et al*, 2003; Wey *et al*, 2005). The results of these investigations suggested a trend toward an association between expression of VEGF and LN metastasis.

In agreement with our proliferation, migration, and invasion data, we found that the downregulation of FXR inhibited NF-*κ*B DNA-binding activity. Moreover, we found a significant reduction of VEGF secretion on the culture medium of pancreatic cancer by the downregulation of FXR using siRNA transfection. It is well accepted that the expression of VEGF is regulated by NF-*κ*B (Huang *et al*, 2001; Shi *et al*, 2001). Therefore, these results suggest that downregulation of FXR could inhibit cancer cell proliferation, migration, and invasion partly through the downregulation of NF-*κ*B and its target gene, VEGF. However, further in-depth studies are needed to ascertain the precise molecular regulation of FXR and NF-*κ*B and their cross-talks in elucidating the role of FXR in cell proliferation, migration, and invasion of pancreatic cancer cells in animal models and in human pancreatic cancer.

In conclusion, our findings show, for the first time, that FXR overexpression in pancreatic cancer tissues with LN metastasis is associated with poor patient survival. Furthermore, we presented experimental evidence that strongly supports the role of FXR downregulation as antitumour and antimetastatic mechanisms in pancreatic cancer. Therefore, downregulation of FXR could be an effective approach for the inactivation and reduction of NF-*κ*B and its target gene, VEGF, which is likely to result in the inhibition of migration, invasion, and metastasis of pancreatic cancer.

## Figures and Tables

**Table 1:** Clinicopathological characteristics^a^

**Characteristics**	**Group I (LN−)**	**Group II (LN+)**
Age (years)	61±10.1	56.6±9.5
*Gender*		
Male	11	9
Female	6	8
		
*Pathological*		
T classification	T3	T3
N classification	N0	N1
Stage	IIA	IIB
		
*Survival*		
Live	6	0
Dead	11	17
Postoperative survival (months)	23.4±8.8	9.1±3.9

Abbreviation: LN=lymph node.

a These patients were selected in a blinded manner.

**Table 2:** Representative upregulated genes that were >1.5-fold expressed in pancreatic cancer tissues with lymph node metastasis

**Probe_ID**	**GB_accession**	**Gene_symbol**	**Gene_name**	**Chromosome**	**Fold (G II/G I)**
200574	BC063443	*LZIC*	*Leucine zipper and CTNNBIP1 domain containing*	1p36.22	3.06
207614	BC035654	*FXR*	*Farnesoid X receptor*	12q23.3	2.63
101570	BC034048	*SCAMP1*	*Secretory carrier membrane protein 1*	5q13.3–q14.1	2.47
184216	BC027956	*SULT1E1*	*Sulphotransferase family 1E, oestrogen-preferring, member 1*	4q13.1	2.32
211511	BC052977	*TNFRSF1B*	*Tumour necrosis factor receptor superfamily, member 1B*	1p36.3–p36.2	1.87
176066	BC022870	*IFI44*	*Interferon-induced protein 44*	1p31.1	1.74
223097	CR457297	*SAV1*	*Salvador homolog 1 (Drosophila)*	14q13–q23	1.71
189219	AL137764	*SMAP1L*	*Stromal membrane-associated protein 1 like*	1p35.3–p34.1	1.70
130750	BC007932	*C1orf165*	*Chromosome 1 open reading frame 165*	1p33	1.68
169562	AF034780	*EDG5*	*Endothelial differentiation, sphingolipid G-protein-coupled receptor, 5*	19p13.2	1.61
208619	BC038505	*BAG4*	*BCL2-associated athanogene 4*	8p11.23	1.57
197126	BX571754	*SERPINB8*	*Serpin peptidase inhibitor, clade B (ovalbumin), member 8*	18q21.3	1.56
188749	AK074638	*ZCCHC8*	*Zinc-finger, CCHC domain containing 8*	12q24.31	1.56
196290	BC005197	*MBIP*	*MAP3K12 binding inhibitory protein 1*	14q13.2	1.54
107454	U90920	*ARHGAP29*	*Rho GTPase-activating protein 29*	1p22.1	1.50
